# Free‐running 3D whole‐heart T_1_ and T_2_ mapping and cine MRI using low‐rank reconstruction with non‐rigid cardiac motion correction

**DOI:** 10.1002/mrm.29449

**Published:** 2022-10-05

**Authors:** Andrew Phair, Gastão Cruz, Haikun Qi, René M. Botnar, Claudia Prieto

**Affiliations:** ^1^ School of Biomedical Engineering and Imaging Sciences King's College London London UK; ^2^ School of Biomedical Engineering ShanghaiTech University Shanghai China; ^3^ Instituto de Ingeniería Biológica y Médica Pontificia Universidad Católica de Chile Santiago Chile; ^4^ Escuela de Ingeniería Pontificia Universidad Católica de Chile Santiago Chile; ^5^ Millennium Institute for Intelligent Healthcare Engineering Santiago Chile

**Keywords:** 3D radial, cine imaging, free‐running, joint $T_{1}/T_{2}$ mapping, low‐rank inversion, non‐rigid motion correction

## Abstract

**Purpose:**

To introduce non‐rigid cardiac motion correction into a novel free‐running framework for the simultaneous acquisition of 3D whole‐heart myocardial $T_{1}$ and $T_{2}$ maps and cine images, enabling a ∼3‐min scan.

**Methods:**

Data were acquired using a free‐running 3D golden‐angle radial readout interleaved with inversion recovery and $T_{2}$‐preparation pulses. After correction for translational respiratory motion, non‐rigid cardiac‐motion‐corrected reconstruction with dictionary‐based low‐rank compression and patch‐based regularization enabled 3D whole‐heart $T_{1}$ and $T_{2}$ mapping at any given cardiac phase as well as whole‐heart cardiac cine imaging. The framework was validated and compared with established methods in 11 healthy subjects.

**Results:**

Good quality 3D $T_{1}$ and $T_{2}$ maps and cine images were reconstructed for all subjects. Septal $T_{1}$ values using the proposed approach (1200±50 ms) were higher than those from a 2D MOLLI sequence (1063±33 ms), which is known to underestimate $T_{1}$, while $T_{2}$ values from the proposed approach (51±4 ms) were in good agreement with those from a 2D GraSE sequence (51±2 ms).

**Conclusion:**

The proposed technique provides 3D $T_{1}$ and $T_{2}$ maps and cine images with isotropic spatial resolution in a single ∼3.3‐min scan.

**Abbreviations**
ADMM: alternating direction method of multipliers
CG: conjugate gradient
CMR: cardiac magnetic resonance
ECG: electrocardiogram
EDV: end‐diastolic volume
EF: ejection fraction
ESV: end‐systolic volume
HD‐PROST: high‐dimensionality undersampled patch‐based reconstruction
IR: inversion recovery
LGE: late gadolinium enhancement
LRI: low‐rank inversion
LRMC: low‐rank motion‐corrected
LV: left ventricular
MRF: magnetic resonance fingerprinting
NUFFT: nonuniform fast Fourier transform

## 1 INTRODUCTION

Cardiac magnetic resonance (CMR) is an important imaging modality that allows for the noninvasive assessment of myocardial disease and cardiac function.^1^, ^2^, ^3^ Ideally, a cardiac exam would enable comprehensive tissue characterization with whole‐heart coverage in an easy‐to‐plan and relatively short (10–15‐min) scan by simultaneously providing multiple imaging outputs such as magnetic relaxation parameter maps (e.g., $T_{1}$ and $T_{2}$ maps), measurements of cardiac function (cine), late gadolinium enhancement (LGE) images and perfusion images, among possible others. In this work, we consider an undersampled respiratory‐ and cardiac‐motion‐corrected simultaneous acquisition of 3D whole‐heart $T_{1}$ and $T_{2}$ maps and cine images at isotropic spatial resolution, pushing CMR closer toward the goal of a short and comprehensive CMR exam.

Established gold standard sequences for 2D myocardial mapping such as MOLLI^4^ ($T_{1}$ mapping) and GraSE^5^ ($T_{2}$ mapping) use data acquired during breath hold and at a single cardiac phase via electrocardiogram (ECG) triggering. In these methods, parameter maps are generated independently by fitting a series of weighted images to exponential relaxation curves on a pixel‐ or voxel‐wise basis.

Methods that jointly acquire both $T_{1}$ and $T_{2}$ myocardial maps in a single breath hold,^6^, ^7^ or utilize rigid respiratory motion correction in a free‐breathing framework,^8^, ^9^ have also been proposed. Such simultaneous acquisitions ensure the parameter maps are inherently co‐registered and reduce the effect of one parameter on the estimation of the other. Similarly, magnetic resonance fingerprinting (MRF)^10^, ^11^ has been applied to 2D myocardial $T_{1}$ and $T_{2}$ mapping in a single breath hold.^12^

The reliance of each of these methods on ECG triggering generally means they are limited to providing maps at a set cardiac phase (e.g., diastole) and exhibit reduced scan efficiency relative to free‐running sequences.

Single‐breath‐hold simultaneous 2D $T_{1}$ mapping and cine imaging has been achieved through the use of continuous data acquisition and the retrospective sorting of the acquired data to cardiac phases.^13^, ^14^, ^15^, ^16^ MRF, together with advanced reconstruction and motion correction techniques, has allowed the extension of simultaneous 2D $T_{1}$ mapping and cine imaging to include $T_{2}$ mapping,^17^, ^18^ and enabled multi‐parametric ECG‐triggered approaches to utilize larger cardiac windows.^19^, ^20^

Jaubert et al.^18^ and Cruz et al.^19^ formulated their low‐rank inversion (LRI)^21^ reconstruction as the minimization of a data consistency term subject to regularization;^22^ a low‐rank dictionary‐compression matrix^21^, ^23^ was incorporated into the encoding operator of the data consistency term, and high‐dimensionality undersampled patch‐based reconstruction (HD‐PROST)^24^ regularization, which enforces the low‐rank property of a tensor constructed from similar multidimensional patches to that centered at each pixel, was employed. Additionally, non‐rigid cardiac motion fields,^16^, ^25^ estimated from auxiliary motion‐resolved reconstructions, have been included in the encoding operator,^19^ utilizing all available data in the reconstruction of each cardiac phase.

The novel multitasking framework^26^, ^27^ has also been proposed for $T_{1}$ and $T_{2}$ mapping across multiple cardiac phases, via the reconstruction of a low‐rank multidimensional image tensor that includes both respiratory‐ and cardiac‐motion dimensions.

The studies considered thus far have mostly considered 2D slices, usually with slice thickness ≥ 8 mm. Hence, they do not provide isotropic whole‐heart coverage and may be subject to through‐plane motion artifacts.

Various approaches for 3D myocardial mapping with whole‐heart coverage have been proposed,^28^, ^29^, ^30^, ^31^, ^32^, ^33^, ^34^, ^35^, ^36^ including joint $T_{1}$ and $T_{2}$ methods,^32^, ^33^, ^34^, ^35^ which utilize many of the innovations previously described for 2D methods, such as MRF,^32^ translational respiratory motion correction,^32^, ^33^, ^35^ low‐rank reconstruction,^32^ and HD‐PROST regularization.^32^, ^33^ While achieving whole‐heart coverage, most of these approaches still use anisotropic spatial resolution (slice thickness ≥8 mm) and do not simultaneously provide cine images like their 2D counterparts.^17^, ^18^

Recently, Qi et al.^37^ reported the first framework to simultaneously provide 3D myocardial $T_{1}$ and $T_{2}$ maps and cine images with isotropic spatial resolution and whole‐heart coverage using LRI with HD‐PROST reconstruction. In that study, interleaved inversion recovery (IR) and $T_{2}$‐preparation ($T_{2}$‐prep) pulses were combined with a free‐running 3D golden‐angle radial acquisition and translational respiratory motion correction, and 3D $T_{1}$ and $T_{2}$ maps and cine images with 2‐mm isotropic spatial resolution were achieved. However, since the k‐space spokes were sorted into cardiac phases using ECG gating, only a fraction of the acquired data was used in the reconstruction of each phase, resulting in a relatively long scan time of ∼11 min to ensure sufficient image quality.

To significantly reduce the scan time, we propose to incorporate non‐rigid cardiac motion correction^19^, ^25^ into the free‐running 3D radial framework that enabled simultaneous cine and whole‐heart mapping.^37^ This new approach allows all acquired data to be used at each cardiac phase. Hence, less overall data with a shorter scan time are able to achieve comparable image quality. The proposed method is evaluated in 11 healthy subjects, and comparable image quality to the LRI with HD‐PROST reconstruction is found using only the first 30% of acquired radial spokes. This corresponds to a simultaneous acquisition of 3D whole‐heart $T_{1}$ and $T_{2}$ maps and cine images in ∼3.3 min.

## 2 METHODS

### 2.1 Acquisition sequence

**FIGURE 1:**
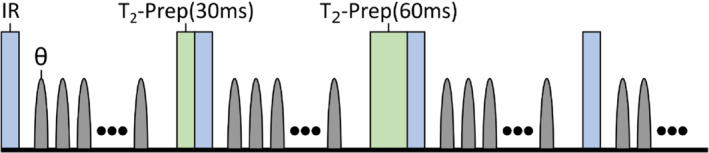
Schematic of the repeating free‐running acquisition sequence. Each shot (duration: 2200 ms) is preceded by an inversion recovery (IR) pulse (blue) and (for two shots in each three‐shot cycle) a $T_{2}$‐preparation ($T_{2}$‐prep) pulse (green). Each gray pulse represents a spoiled gradient echo readout with flip angle θ and k‐space orientation determined by a 3D golden‐angle sampling scheme.

Data were acquired using a low‐flip‐angle spoiled gradient echo readout with a 3D golden‐angle radial trajectory, interleaved with IR and $T_{2}$‐prep pulses to provide sufficient $T_{1}$ and $T_{2}$ encoding, as introduced by Qi et al.^37^ The three‐shot cycle seen in Figure 1 was repeated throughout the scan. Each shot was preceded by an IR pulse, alongside, for two of the shots, a $T_{2}$‐prep pulse with an echo time of 30 or 60 ms.

### 2.2 Reconstruction framework

**FIGURE 2:**
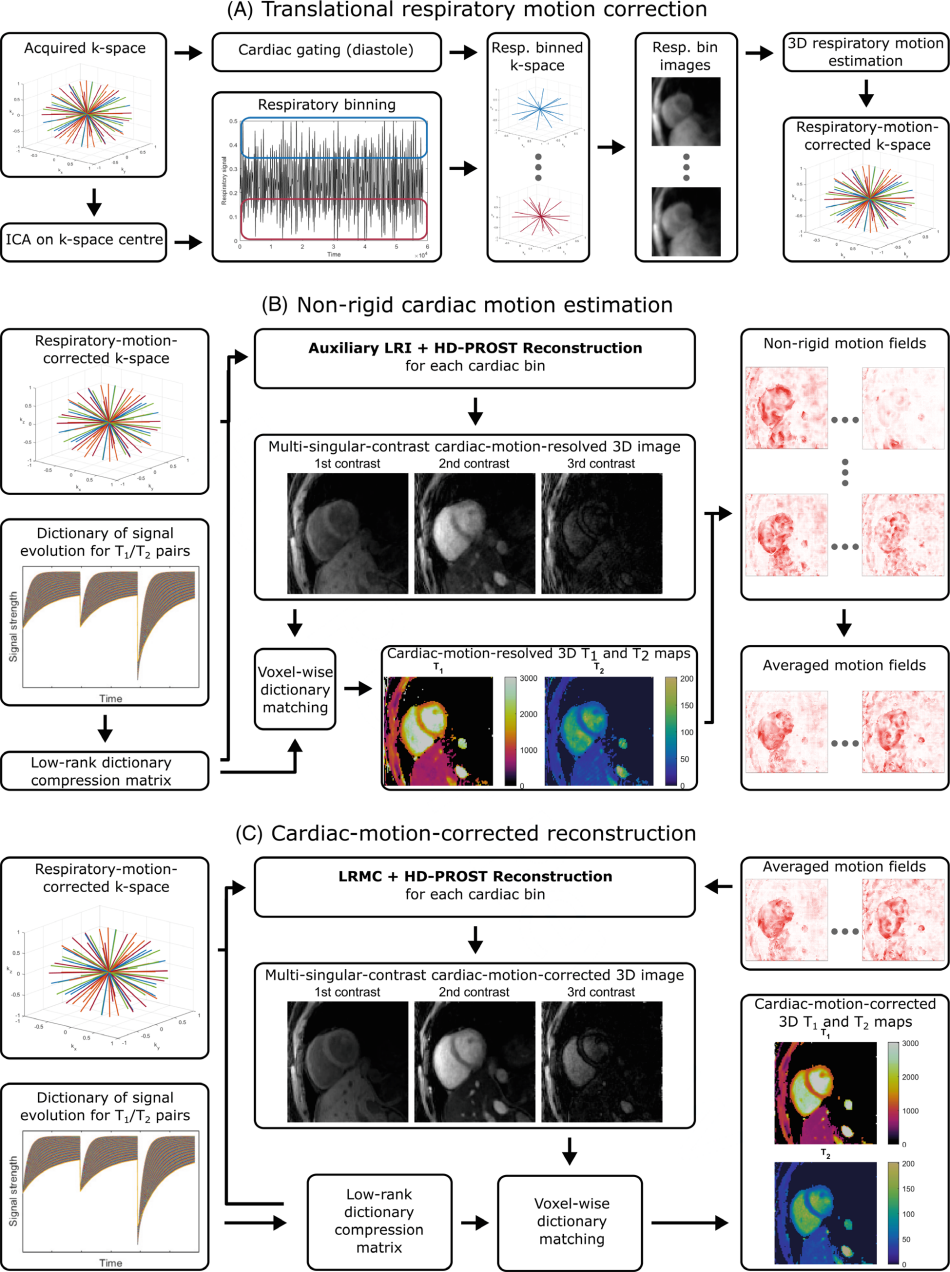
Schematic of the proposed reconstruction framework that consists of three primary steps: (A) The estimation of, and correction for, translational respiratory motion, (B) the estimation of the non‐rigid cardiac motion from motion‐resolved LRI+HD‐PROST reconstructions, and (C) the iterative low‐rank motion‐corrected (LRMC) reconstruction with HD‐PROST regularization.

The proposed reconstruction framework consisted of three main steps: (1) translational respiratory motion correction; (2) estimation of non‐rigid cardiac motion from motion‐resolved low‐rank inversion (LRI)^21^ reconstructions; and (3) cardiac‐motion‐corrected reconstruction with patch‐based low‐rank regularization (HD‐PROST).^24^ The flow diagram of the proposed reconstruction framework is presented in Figure 2. Details of each step are given below.

#### 2.2.1 Translational respiratory motion correction

Independent component analysis was applied to the k‐space center (acquired in every spoke) to extract a 1D respiratory signal.^36^, ^38^, ^39^ As shown in Figure 2A, this signal was then used to sort spokes into five respiratory bins, while the synchronized ECG signal was used to exclude all spokes acquired outside a temporal window centered at diastole. An auxiliary self‐calibrated GROG^40^ reconstruction was performed for each bin, and a manually selected volume surrounding the heart was extracted for translational motion registration with the in‐built geometric transformation function in MATLAB (The Mathworks, Natick, USA). For the dth bin, a 3D translation vector $T_{d}=T_{xd}\hat{x}+T_{yd}ŷ+T_{zd}\hat{z}$ was estimated, representing the respiratory shift between that bin and the end‐expiration reference bin. A unique translation vector for each k‐space spoke was derived by linearly interpolating those vectors corresponding to the nearest bin centers on either side of the spoke's respiratory signal value. k‐Space spokes with a respiratory signal outside the minimum and maximum bin‐center values were assigned the translation vector of the nearest bin center. Finally, each k‐space spoke was corrected for translational respiratory motion by applying the phase shift

(1) $${\hat{b}}_{jm}=b_{jm}\exp\left(2\pi ik_{jm}\cdot T_{m}\right),$$

where $b_{jm}$ is the uncorrected value of the k‐space sample at position j along the mth spoke, ${\hat{b}}_{jm}$ is the corrected value, $k_{jm}$ is the 3D position vector representing the k‐space location of that sample, and $T_{m}$ is the translation vector for the mth spoke.

#### 2.2.2 Non‐rigid cardiac motion estimation

Auxiliary cardiac‐motion‐resolved images were reconstructed using the LRI approach with HD‐PROST regularization to estimate 3D non‐rigid cardiac motion fields. These reconstructions are equivalent to those previously reported by Qi et al.,^37^ and function both as a comparison to the proposed technique and as the input to estimate the non‐rigid cardiac motion estimation.

Alongside the respiratory‐motion‐corrected k‐space data, the LRI+HD‐PROST reconstruction required the input of a dictionary‐compression matrix, coil sensitivity maps, and sampling‐density compensation weights.

The $N_{t}\times N_{D}$ dictionary D was created by simulating the signal evolution for $N_{D}$ different combinations of $T_{1}$ and $T_{2}$ values in the physiological range at $N_{t}$ discrete time points corresponding to the spoke‐acquisition times in the sequence. An SVD of the dictionary yields $D=USV^{*}$, where $U\in {\mathbb{C}}^{N_{t}\times N_{t}}$ and $V\in {\mathbb{C}}^{N_{D}\times N_{D}}$ are unitary matrices, $S\in {\mathbb{R}}^{N_{t}\times N_{D}}$ is a diagonal matrix containing the singular values of D in descending order, and ∗ denotes the conjugate transpose. If $U_{r}$ is defined as a sub‐matrix of U containing only the first r columns, then its conjugate transpose $U_{r}^{*}$ forms the dictionary‐compression matrix, which compresses the dictionary D such that each entry now contains the first r singular contrasts instead of every acquired contrast.

LRI with HD‐PROST regularization was employed to reconstruct each cardiac phase by minimizing

(2) $$\begin{array}{ll} {ℒ}_{\text{LRI}}\left(\rho ,𝒯,Y\right) & ={\left\|E_{q}\rho -W_{q}k_{q}\right\|}_{2}^{2}+\lambda \underset{p}{\sum }{\left\|{𝒯}_{p}\right\|}_{*}\\ & \;+\frac{\mu }{2}\underset{p}{\sum }{\left\|{𝒯}_{p}-P_{p}\left(\rho \right)-P_{p}\left(Y\right)\right\|}_{F}^{2}. \end{array}$$

Here, for N voxels, $N_{c}$ coils, r singular contrasts, and $K_{q}$ k‐space samples at the qth cardiac phase, $\rho \in {\mathbb{C}}^{Nr\times 1}$ is the multi‐singular‐contrast 3D image vector being solved for; $k_{q}\in {\mathbb{C}}^{K_{q}N_{c}\times 1}$ is the vector of translational‐motion‐corrected multi‐coil k‐space data at the qth cardiac phase; $W_{q}\in {\mathbb{R}}^{K_{q}N_{c}\times K_{q}N_{c}}$ is a diagonal matrix of radial sampling‐density compensation weights; ${𝒯}_{p}$ is the HD‐PROST tensor of patches similar to the patch centered at the pth voxel; $P_{p}$ is a patch selection operator; Y is the augmented Lagrangian; λ and μ are penalty weightings; ∗ and F denote the nuclear and Frobenius norms, respectively; and

(3) $$E_{q}=W_{q}U_{rq}F_{q}S$$

is the encoding operator, which consists of $S\in {\mathbb{C}}^{NN_{c}r\times Nr}$, the estimated 3D coil sensitivity maps, $F_{q}\in {\mathbb{C}}^{K_{q}N_{c}r\times NN_{c}r}$, the nonuniform Fourier operator that transforms the 3D image of each coil and each singular contrast to a corresponding 3D radial k‐space with only those spokes acquired at the qth cardiac phase, and $U_{rq}\in {\mathbb{R}}^{K_{q}N_{c}\times K_{q}N_{c}r}$, which contains the dictionary‐compression weights that relate the singular contrasts to the $T_{1}$ and $T_{2}$ weighting of the contrast that each spoke (in the qth phase) was acquired with.

Equation (2) was solved by employing the alternating direction method of multipliers (ADMM)^41^ technique, alternating between satisfying the first (data consistency) term via conjugate gradient (CG) descent and enforcing the low‐rank property of the HD‐PROST tensors ${𝒯}_{p}$, as described in detail by Bustin et al.^24^

To accelerate the iterative reconstruction, the Toeplitz approach^42^, ^43^, ^44^, ^45^, ^46^ was employed. This allowed the many computationally expensive nonuniform fast Fourier transform (NUFFT) operations, which arose in each CG iteration due to the multiplication of the image‐guess by $E_{q}^{*}E_{q}$, to be replaced with efficient 3D FFT operations on a grid with dimensions equal to twice the image size. The requisite initial evaluation of $E_{q}^{*}\left(W_{q}k_{q}\right)$ was achieved using NUFFT^47^ operations once and then saved.

The auxiliary cardiac‐motion‐resolved reconstructions were then used to estimate 3D non‐rigid cardiac motion fields using the software NiftyReg.^48^ The motion fields were found to be highly sensitive to the contrast of the image used in registration. As such, four separate sets of fields were estimated, using the first and second singular‐contrast images and using $T_{1}$ and $T_{2}$ maps generated from the auxiliary cardiac‐motion‐resolved images via dictionary matching (Figure 2B). The final motion fields were obtained by averaging the displacement vectors at each voxel location. If the vector with the greatest displacement from the average had a displacement greater than two voxel side lengths, it was considered an outlier and excluded from the average.

#### 2.2.3 Motion‐corrected reconstruction

Incorporating the estimated non‐rigid cardiac motion fields into the reconstruction alongside the low‐rank dictionary compression operator and HD‐PROST regularization allowed all the (respiratory‐motion‐corrected) k‐space data to be utilized in the reconstruction of each cardiac phase (Figure 2C), as in the manner of the previous low‐rank motion‐corrected (LRMC) study.^19^ Since the motion fields capture the underlying physiological motion, they can be applied to any time‐point or singular‐contrast image. Here, it was computationally advantageous to apply the motion distortion to each contrast in the multi‐singular‐contrast image ρ before multiplication by the coil sensitivity maps. A mathematical derivation justifying this order of operations is included in the supporting information online. Consequently, the cost function given in Equation (2) was modified to become

(4) $$\begin{array}{ll} {ℒ}_{\text{LRMC}}\left(\rho ,𝒯,Y\right) & ={\left\|E\rho -Wk\right\|}_{2}^{2}+\lambda \underset{p}{\sum }{\left\|{𝒯}_{p}\right\|}_{*}\\ & \;+\frac{\mu }{2}\underset{p}{\sum }{\left\|{𝒯}_{p}-P_{p}\left(\rho \right)-P_{p}\left(Y\right)\right\|}_{F}^{2}. \end{array}$$

Here, where K is the total number of k‐space samples, $k\in {\mathbb{C}}^{KN_{c}\times 1}$ is the vector of translational‐motion‐corrected multi‐coil k‐space data across all cardiac phases; $W\in {\mathbb{R}}^{KN_{c}\times KN_{c}}$ contains the density compensation weights for all samples;

(5) $$E=\underset{q}{\sum }WU_{r}A_{q}F_{q}SM_{q}$$

is the encoding operator that now sums across all cardiac phases and incorporates $M_{q}\in {\mathbb{R}}^{Nr\times Nr}$, which acts to apply the motion distortion for the qth cardiac phase, $A_{q}\in {\mathbb{N}}^{KN_{c}r\times K_{q}N_{c}r}$, an operator that places the k‐space spokes from each cardiac phase into the correct positions in the full radial trajectory, and $U_{r}\in {\mathbb{R}}^{KN_{c}\times KN_{c}r}$, which contains the dictionary‐compression weights for all spokes. All other variables are defined as they were previously.

#### 2.2.4 $T_{1}$ and $T_{2}$ mapping

Three‐dimensional $T_{1}$ and $T_{2}$ maps were obtained from the output cardiac‐motion‐corrected multi‐singular‐contrast image at any given cardiac phase via voxel‐wise dictionary matching (Figure 2C). The closest match for each voxel was determined as the highest‐valued dot product, and the $T_{1}$ and $T_{2}$ values used in the generation of that dictionary entry were assigned to the corresponding position in the 3D maps.

#### 2.2.5 Cine imaging

Three‐dimensional cine images were reconstructed by solving Equation (4) for every cardiac phase, yielding multiphase multi‐singular‐contrast 3D images. The second singular contrast was selected for display within the paper since it was seen to exhibit good contrast between the myocardium and the blood pool.

### 2.3 In vivo imaging

Eleven healthy subjects (eight female) were recruited with approval from the local institutional review board and written informed consent was given by all subjects prior to scanning.

The proposed free‐running sequence was implemented for all subjects on a 1.5‐T scanner (Ingenia, Philips Healthcare, Best, Netherlands) using a 28‐channel cardiac coil and the following imaging parameters: FOV (with two‐fold readout oversampling) = 200 mm × 200 mm × 200 mm, spatial resolution = 2 mm × 2 mm × 2 mm, flip angle = 6°, spokes per shot = 195, shot interval = 2200 ms, TR = 10.3 ms, TE = 4.6 ms, $T_{\text{gap}}$ = 9.5 ms (time between preparatory pulses and first readout), $T_{\text{ex}}$ = 182 ms (recovery time between last readout and next preparatory pulse).

A “full” scan consisted of 300 shots and thus had an 11‐min scan time. Any subsequent undersampling was performed retrospectively by utilizing only the first shots acquired in the sequence.

For comparison, 2D MOLLI ($T_{1}$ maps) and 2D GrASE ($T_{2}$ maps) scans at diastole and conventional 2D cine scans were performed, each in the mid‐short‐axis slice orientation.

The conventional 2D cine scan parameters were as follows: FOV = 288 mm × 288 mm, spatial resolution = 1.5 mm × 1.5 mm, slice thickness = 10 mm, flip angle = 60°, TR = 2.9 ms, TE = 1.4 ms, 16 cardiac phases.

The 2D MOLLI (3‐3‐5) parameters were as follows: FOV = 288 mm × 288 mm, spatial resolution = 1.5 mm × 1.5 mm, slice thickness = 8 mm, flip angle = 35°, TR = 2.6 ms, TE = 1.3 ms, diastolic acquisition window = 187 mm.

The 2D GraSE parameters were as follows: FOV = 288 mm × 288 mm, spatial resolution = 2 mm × 2 mm, slice thickness = 8 mm, flip angle = 90°, TR = 1 heartbeat, ΔTE = 8.8 ms, turbo spin‐echo factor = 9, echo‐planar‐imaging readout factor = 7, double IR black‐blood prepulse.

Additionally, multi‐slice 2D cine scans were performed for five subjects with short‐axis slices covering the left ventricle. Parameters for these scans included: FOV = 320 mm × 320 mm, spatial resolution = 0.95 mm × 0.95 mm, slice thickness = 8 mm, 12 slices, flip angle = 60°, TR = 3.0 ms, TE = 1.5 ms, 20 cardiac phases.

### 2.4 Reconstructions

LRI (cardiac‐motion‐resolved) and LRMC (cardiac‐motion‐corrected) reconstructions at the diastolic phase were implemented for two subjects with and without HD‐PROST regularization and using four different amounts of data corresponding to different effective scan times: 100% of acquired data (58 500 spokes, 11 min), 50% (29 250 spokes, 5.5 min), 30% (17 550 spokes, 3.3 min), and 20% (11 700 spokes, 2.2 min). We note that LRI+HD‐PROST reconstructions at every phase were required for the estimation of the motion fields used in the LRMC+HD‐PROST reconstruction at diastole. For both reconstructions, r=3 singular contrasts were utilized and data were assigned to 16 cardiac phases.

Analysis of these results (reported below) led to the choice of 30% of the spokes as our proposed acceleration. Hence, for all subjects, LRI and LRMC reconstructions with HD‐PROST were implemented using 100% and 30% of the acquired data at all phases, allowing both diastolic parameter mapping and 3D cine imaging.

Parameters of the NiftyReg^48^ motion field estimation were as follows: number of levels = 3, grid spacing = 2, standard deviation of Gaussian kernel = 3, regularization penalty weights = 0.005 (bending energy), 0.0002 ($L^{2}$ displacement norm), and 0.01 (Jacobian determinant). Parameters of the HD‐PROST regularization were as follows: patch size = 5 × 5 × 5, maximum number of similar patches = 20, search window size = 20 × 20 × 20, λ=750, μ=0.02. Parameters of the ADMM iteration were as follows: number of ADMM iterations = 4, number of CG iterations per ADMM iteration (LRI) = 9, number of CG iterations per ADMM iteration (LRMC) = 3.

Dictionary entries were calculated at the $N_{t}=385$ discrete time points corresponding to the 195 spoke‐acquisition times in each of the three shots. A total of 138 $T_{1}$ values (between 200 and 3000) and 303 $T_{2}$ values (between 10 and 200) were considered, resulting in $N_{D}=41\;814$ total entries.

Coil sensitivity maps were estimated for each cardiac phase using Walsh's method.^49^

### 2.5 Analysis

#### 2.5.1 3D $T_{1}$ and $T_{2}$ maps

The mean and standard deviation of septal parameter values in a given map were determined by manually segmenting the septum in mid‐short‐axis slices and considering the values of pixels located in that region.

Further analysis of the 3D parameter maps was performed via the manual segmentation of the left ventricular (LV) wall into the 16 segments of the American Heart Association (AHA) standard.^50^ Segmentation was performed on all short‐axis slices covering the LV wall, with segment means and standard deviations for basal, mid‐ventricular, and apical segments calculated over an appropriate range of slices.

#### 2.5.2 Cine images

End‐diastolic volume (EDV), end‐systolic volume (ESV), and ejection fraction (EF) measurements were used to compare the 3D cine images from different amounts of acquired data and the multi‐slice 2D cine images (where those scans were available).

EDV and ESV values were determined via a manual slice‐by‐slice segmentation of the left ventricle at diastole and systole, respectively. The EF was then calculated as

(6) $$\text{EF}=\frac{\text{EDV}-\text{ESV}}{\text{EDV}}\cdot 100\%.$$

## 3 RESULTS

**FIGURE 3:**
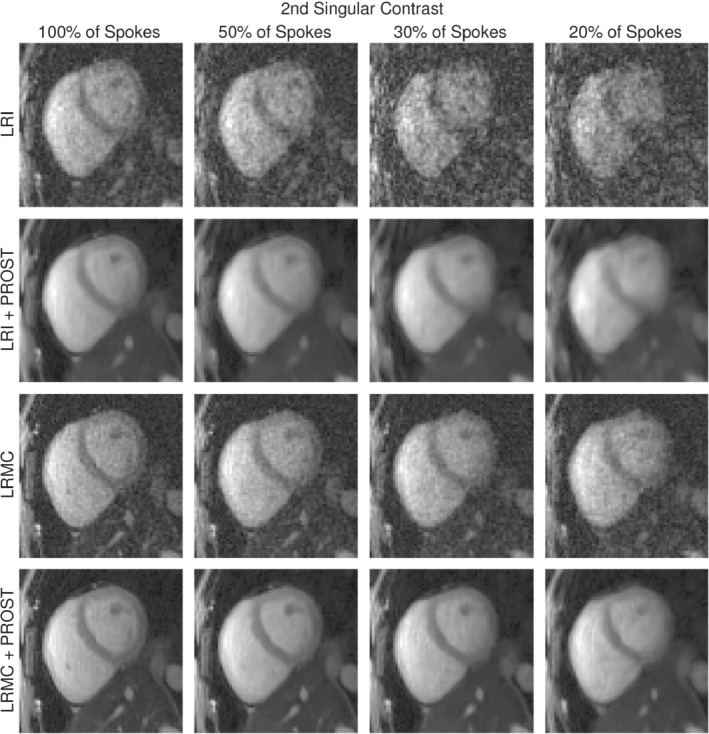
Mid‐ventricular short‐axis slices of the 3D second‐singular‐contrast images produced by different reconstruction methods and utilizing different amounts of k‐space data for one representative subject.

**FIGURE 4:**
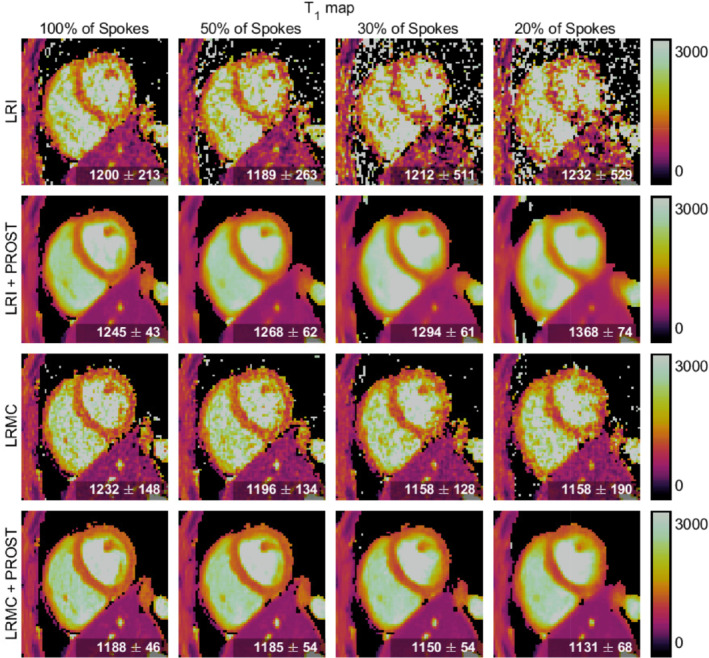
Mid‐ventricular short‐axis slices of the 3D $T_{1}$ maps produced by different reconstruction methods and utilizing different amounts of k‐space data for one representative subject. The mean and standard deviation of the $T_{1}$ value in the septum region are given in the corner of each panel.

**FIGURE 5:**
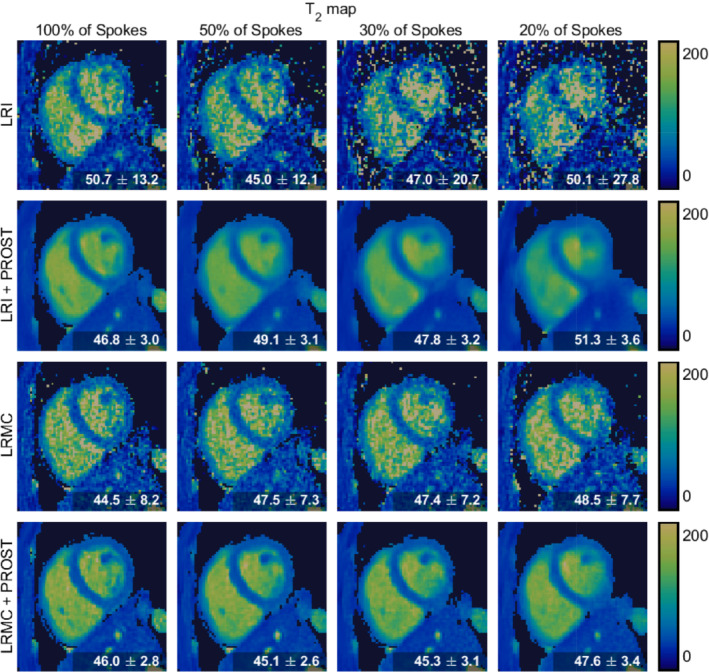
Mid‐ventricular short‐axis slices of the 3D $T_{2}$ maps produced by different reconstruction methods and utilizing different amounts of k‐space data for one representative subject. The mean and standard deviation of the $T_{2}$ value in the septum region are given in the corner of each panel.

Figures 3, 4, 5 display a diastolic mid‐short‐axis slice of the second singular‐contrast images, $T_{1}$ maps and $T_{2}$ maps, respectively, for one representative subject, produced using the LRI (cardiac‐motion‐resolved) and LRMC (cardiac‐motion‐corrected) reconstruction schemes both with and without HD‐PROST regularization and with varying percentages of the total acquired data. In all cases, the reconstructions without HD‐PROST exhibit noise‐like artifacts that tend to increase in severity with increased undersampling. Comparing the images and maps achieved with HD‐PROST at each data percentage visually, the motion‐corrected reconstructions appear to have sharper features, notably around the septum and papillary muscles. As the number of spokes incorporated in the reconstruction is reduced from 100% to 20%, the quality of the LRI images and maps significantly decreases and blurring becomes evident, whereas the LRMC reconstruction appears to be more robust.

These observations are consistent with the mean and standard deviation of the parameter values within the septum, as reported in the bottom corner of each $T_{1}$ and $T_{2}$ map (Figures 4 and 5, respectively).

The standard deviation of the $T_{1}$ septum values tends to increase for each reconstruction method as the amount of data utilized decreases. For the LRI reconstructions without HD‐PROST, a standard deviation of 213 ms is recorded with 100% of acquired data, increasing to 529 ms with 20% of data. For LRMC without HD‐PROST, this is reduced to an increase from 148 to 190 ms. The application of HD‐PROST further increases the precision, with the LRMC+HD‐PROST reconstructions recording standard deviations between 48 ms (100% of data) and 68 ms (20%). Additionally, the mean septum $T_{1}$ values increase to values in the range of 1200–1400 ms for both LRI reconstructions at low data percentages, whereas the means recorded with the LRMC reconstructions remain in the 1100–1250 ms range.

Similar trends are evident in the $T_{2}$ maps, and although the LRI+HD‐PROST reconstructions here appear more robust to undersampling, they are still outperformed by LRMC+HD‐PROST. The standard deviation of the $T_{2}$ septum values increases from 13.2 to 27.8 ms with decreasing acquired data for the LRI reconstructions without HD‐PROST and is in the range of 7–9 ms for all LRMC reconstructions without HD‐PROST. Comparatively, the standard deviation of ∼3 ms recorded for both LRI+HD‐PROST and LRMC+HD‐PROST reconstructions using 100% of the acquired data is maintained down to 20% of the data.

Singular‐contrast images and parameter maps for a second subject are presented in Supporting Information Figures S1–S3 (available online) and display similar trends.

Balancing the desire to minimize the amount of acquired data, and hence the effective scan time, with the consequent reduction in image quality, we select an undersampling to 30% of the acquired spokes (equivalent to a 3.3‐min scan time) as our proposed acceleration for further evaluation of the LRMC method. Note that HD‐PROST is utilized in all the results that follow.

### 3.1 $T_{1}$ and $T_{2}$ mapping

**FIGURE 6:**
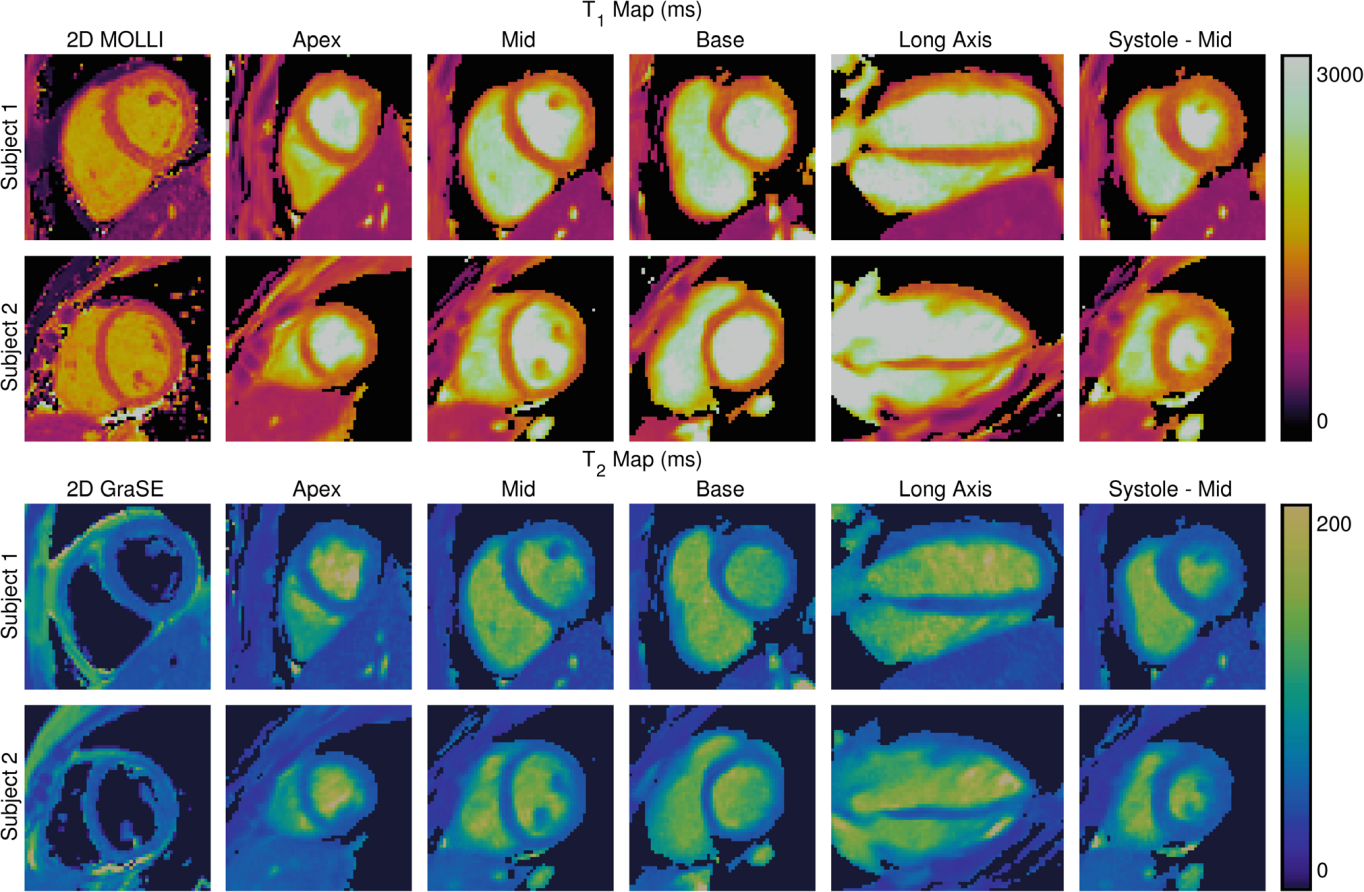
Comparison of the $T_{1}$ maps (top) and $T_{2}$ maps (bottom) acquired using the established 2D mapping sequences MOLLI ($T_{1}$) and GraSE ($T_{2}$) and the proposed approach with 30% of the acquired data for two representative subjects. The MOLLI and GraSE maps are acquired for a diastolic mid‐short‐axis slice, whereas for the proposed approach, three diastolic short‐axis slices (apex, mid, and base) are displayed, alongside a diastolic vertical‐long‐axis slice and a systolic mid‐short‐axis slice.

Diastolic $T_{1}$ and $T_{2}$ maps for two representative subjects reconstructed using the proposed technique and 30% of the acquired data are presented alongside the 2D maps at mid‐short‐axis produced using the conventional methods MOLLI and GraSE in Figure 6. Since the proposed technique is 3D with isotropic spatial resolution, the maps can be reformatted to depict arbitrary slices and orientations. Here, one vertical long‐axis slice and three short‐axis slices (including mid‐ventricular, for direct comparison) are displayed. Additionally, mid‐ventricular short‐axis maps at systolic phase are presented. Good image quality is observed in the 3D maps, comparable to that of the conventional 2D methods.

**FIGURE 7:**
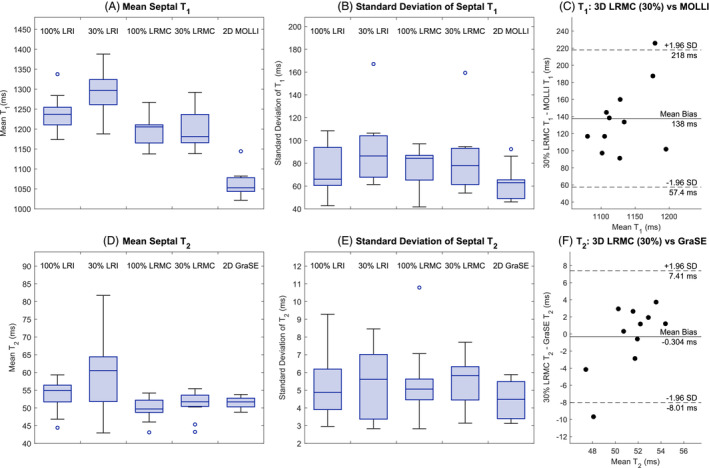
Boxplots of the mean (left) and standard deviation (center) of the $T_{1}$ (top) and $T_{2}$ (bottom) values recorded in the septum region across all 11 subjects using 100% or 30% of the acquired data, and either a motion‐resolved (LRI+HD‐PROST) or motion‐corrected (LRMC+HD‐PROST) reconstruction, or established 2D mapping sequences (MOLLI and GraSE). Bland–Altman plots (right) compare the septal parameter values recorded using the proposed (30% LRMC+HD‐PROST) and conventional (MOLLI and GraSE) methods pairwise.

For a quantitative comparison between the proposed and existing methods, the mean and standard deviation of the $T_{1}$ and $T_{2}$ values in the septum are plotted in Figure 7. Figure 7A shows that the mean $T_{1}$ value for MOLLI (1063±33 ms) is lower than that achieved using the previous LRI+HD‐PROST method with 100% of the acquired data (1237±45 ms) or the proposed LRMC+HD‐PROST method with 30% of the data (1200±50 ms). The Bland–Altman plot in Figure 7C similarly shows a mean bias of +138 ms for the proposed approach relative to MOLLI. However, MOLLI is known to underestimate $T_{1}$ values.^51^ The standard deviation of $T_{1}$ values in each septum region is slightly higher with the 3D approaches (74±23 ms for 100% LRI+HD‐PROST, 83±29 ms for 30% LRMC+HD‐PROST) than with MOLLI (62±15 ms).

The equivalent plots for the $T_{2}$ values (Figure 7D–F ) indicate close agreement between the mean $T_{2}$ values from the proposed 30% LRMC+HD‐PROST approach ( 51±4 ms) and the 2D GraSE sequence ( 51±2 ms), and a difference of 0 ms is seen to be within the 95% confidence interval on the Bland–Altman plot in Figure 7F (mean bias = −0.3 ms, standard deviation = 3.8 ms). Figure 7E indicates that all methods exhibit similar precision, with GraSE (standard deviation = 4.5±1.0 ms) slightly more precise than the LRMC+HD‐PROST technique with 30% of data ( 5.5±1.4 ms).

**FIGURE 8:**
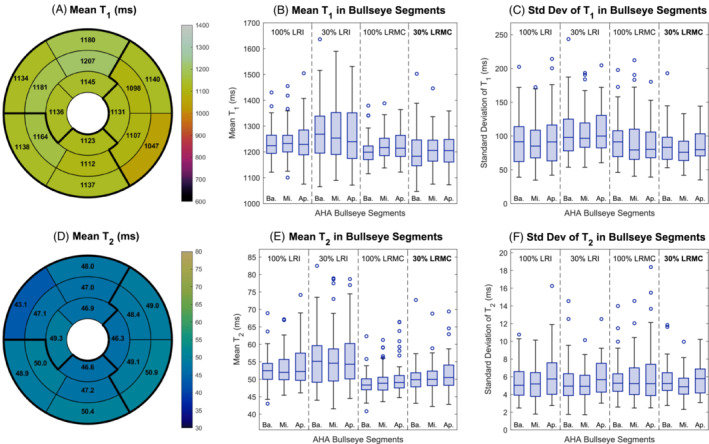
$T_{1}$ (top) and $T_{2}$ (bottom) values recorded in the 16 AHA myocardial segments. Bullseye plots (left) for one representative subject using the LRMC+HD‐PROST approach with 30% of the acquired data. Boxplots of the mean (center) and standard deviation (right) recorded in each bullseye segment in the basal, mid‐ventricular, and apical regions, across all 11 subjects, using 100% or 30% of the acquired data and either a motion‐resolved (LRI+HD‐PROST) or motion‐corrected (LRMC+HD‐PROST) reconstruction.

Bullseye plots of the myocardial $T_{1}$ and $T_{2}$ values in the maps reconstructed using the proposed LRMC+HD‐PROST approach are presented in Figure 8 for one subject and demonstrate that it achieves relatively homogeneous values across the myocardium. Figure 8 also includes boxplots of the mean and standard deviation of the $T_{1}$ and $T_{2}$ segment values over all 11 subjects, for all of the 3D reconstructions considered (LRI+HD‐PROST and LRMC+HD‐PROST with 30% and 100% of the acquired data). The $T_{1}$ standard deviation across all segments and subjects for 30% LRMC+HD‐PROST was 84±24 ms, which compares to 105±32 ms for 30% LRI+HD‐PROST. For $T_{2}$, the standard deviation across all segments and subjects was similar for all four methods: 5.5±2.1 for 100% LRI+HD‐PROST, 5.5±2.0 ms for 30% LRI+HD‐PROST, 5.9±2.7 ms for 100% LRMC+HD‐PROST, and 5.4±1.7 ms for the proposed 30% LRMC+HD‐PROST.

### 3.2 Cine images

**FIGURE 9:**
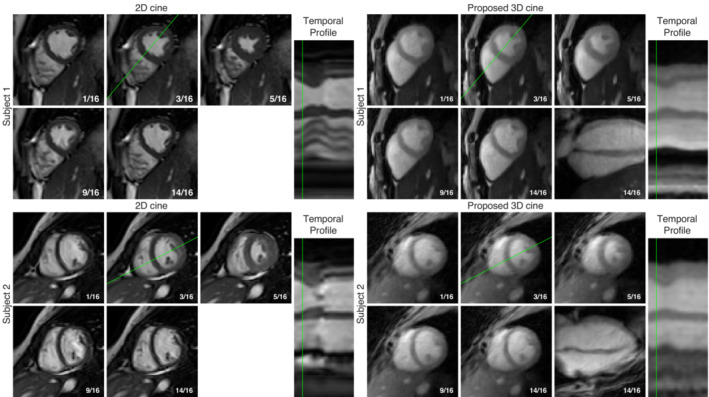
Comparison of the mid‐short‐axis 2D cine with a matching slice from the 3D cine reconstructed using the proposed LRMC+HD‐PROST approach with 30% of the acquired data for two example subjects. Five representative frames of the 16‐frame cine images are shown, alongside a temporal profile displaying the temporal variation of the image along the green line indicated for each cine in the third‐phase panel. An additional vertical‐long‐axis slice at the 14th phase is shown for the 3D cine images.

Frames from the mid‐short‐axis slice of the 3D cine images for two subjects, reconstructed using the proposed approach with 30% of the acquired data, are presented alongside frames from the conventional 2D cine images acquired for those subjects in Figure 9 . Additionally, temporal profiles show the motion over all 16 frames on a line passing through the LV. A video of the 2D and 3D cine images for the first subject is included in the supporting information online (Video S1).

**FIGURE 10:**
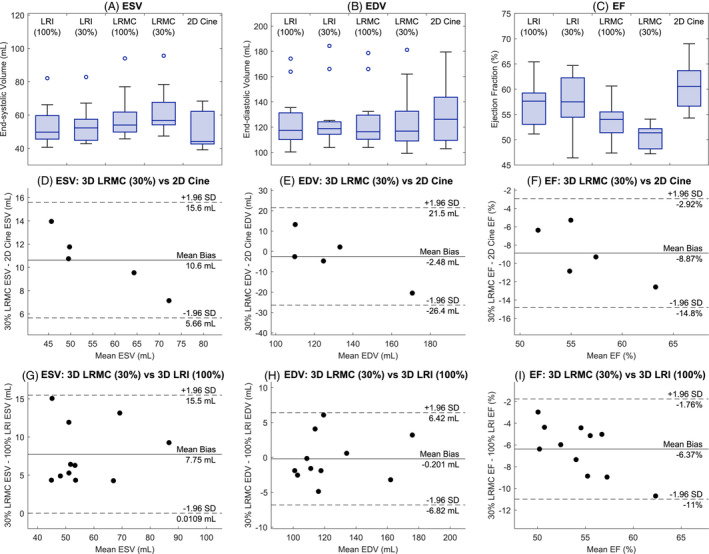
The cardiac function indicators end‐systolic volume (ESV), end‐diastolic volume (EDV), and ejection fraction (EF) as measured from 3D and 2D multi‐slice cine images. (A–C) Boxplots of the recorded values using the 3D radial acquisition, LRI or LRMC (+HD‐PROST) reconstruction and 100% or 30% of the acquired data (11 subjects), and the conventional 2D multi‐slice cine (five subjects). (D–F) Bland–Altman plots comparing the proposed (30% LRMC+HD‐PROST) approach against the 2D multi‐slice cine pairwise (five subjects). (G‐I) Bland–Altman plots comparing the proposed (30% LRMC+HD‐PROST) approach against the previous (100% LRI+HD‐PROST) approach pairwise (11 subjects).

Quantitative indicators of cardiac function are presented in Figure 10, where measurements of ESV, EDV, and EF are plotted for 2D cine (for the five subjects where multi‐slice 2D cines were acquired) and 3D cine (for all 11 subjects), and pairwise comparisons using Bland–Altman plots are shown between the proposed (30% LRMC+HD‐PROST) and multi‐slice 2D techniques and the proposed (30% LRMC+HD‐PROST) and previous (100% LRI+HD‐PROST) techniques. Figure 10C shows that the EF measured from the LRMC+HD‐PROST methods with 30% or 100% of the acquired data (50.6±2.4% and 53.7±3.5%, respectively) is lower than that from the multi‐slice 2D cines (60.6±5.2%) or LRI+HD‐PROST with 30% or 100% of acquired data (57.3±5.4% and 57.1±4.3%, respectively). We note that the higher EF values obtained with 30% LRI+HD‐PROST compared with 30% LRMC+HD‐PROST are despite the poorer quality images and maps produced by this method, as seen in Figures 3, 4, 5. The Bland–Altman plots comparing EF measurements (Figure 10F,I) also reflect the underestimation of EF, with the proposed 30% LRMC+HD‐PROST method recording a mean bias of −8.9% relative to multi‐slice 2D.

## 4 DISCUSSION

We have presented a framework that allows joint 3D whole‐heart myocardial $T_{1}$ and $T_{2}$ mapping and cine imaging with isotropic spatial resolution in a single ∼3‐min scan via the incorporation of non‐rigid cardiac motion correction alongside low‐rank dictionary compression and patch‐based regularization. The scan is easy to plan, free‐breathing, achieves 100% scan efficiency due to its continuous acquisition scheme, and represents a significant step toward an easy‐to‐use and comprehensive whole‐heart CMR exam. The proposed reconstruction method was successfully applied for 11 subjects and compared to the results of conventional 2D mapping sequences and cine images and the previously proposed cardiac‐motion‐resolved method^37^ with an 11‐min scan time.

Good agreement was seen between the myocardial $T_{2}$ values achieved using the proposed technique and the 2D GraSE scans. Higher $T_{1}$ values were observed than with the conventional MOLLI $T_{1}$‐mapping sequence; however, the latter is known to underestimate $T_{1}$. For both $T_{1}$ and $T_{2}$ parameter maps, the standard deviations recorded within the septum (Figure 7) and within individual AHA segments (Figure 8) were slightly higher with the proposed approach than the gold standard 2D sequences, but generally comparable with the 3D technique utilizing more data.

Compared with the conventional multi‐slice 2D cine imaging, the proposed approach was seen to underestimate the EF with a mean bias of −8.9%. One potential cause of this error is the accuracy of the estimated non‐rigid motion fields; the corresponding mean biases evaluated using the same five subjects were −6.2% for 100% LRMC+HD‐PROST and −2.3% for 100% LRI+HD‐PROST. This suggests that without motion correction a more accurate EF estimate is achieved, despite reduced image quality. The Bland–Altman plots of these EF results are included in supporting information Figure S4A‐C. Considering only the motion‐corrected (LRMC) reconstructions, we see that the magnitude of the mean EF bias increases from 6.2% to 8.9% when the amount of k‐space data incorporated is reduced from 100% to 30%. This could be due to a reduction in the accuracy of the motion fields when they are estimated from lower‐quality motion‐resolved images, the reduced amount of data included in the motion‐corrected reconstruction, or a combination of both. Since there are no ground‐truth motion fields to compare against, to explore this effect we instead consider “mixed” motion‐corrected reconstructions that utilize 30% of the acquired data but use the motion fields estimated from the 100% LRI reconstructions. We find that across all 11 subjects, the mean EF bias between 30% LRMC+HD‐PROST and 100% LRMC+HD‐PROST reconstructions of 2.7% is reduced to 0.2% for the “mixed” reconstructions, with ESV bias reduced from 2.4mL to −0.5mL and EDV bias roughly constant at 1.3mL. This suggests that the primary cause of EF underestimation when moving from 100% to 30% of data with the motion‐corrected reconstruction is the quality of the motion fields. Bland–Altman plots of the ESV, EDV, and EF for these reconstructions are included in supporting information Figure S5.

However, motion field quality is not entirely sufficient to explain the bias in cardiac function measurements. Relative to multi‐slice 2D cine, the 100% motion‐resolved LRI+HD‐PROST reconstructions record a mean EF bias of −2.3% (Figure S4C). Since the 2D multi‐slice cine consists of 8‐mm‐thick slices, its ESV and EDV measurements are based on more‐discretized volume segmentations, and in some cases, the most apical or basal slice may not be acquired. A more direct comparison between the 3D and 2D cine images can be performed by estimating ESV and EDV from the 3D reconstructions using only those slices that match the slices in the multi‐slice cine, that is, every fourth 2‐mm‐thick slice. Using this measurement, the 100% LRI+HD‐PROST EF bias is reduced from −2.3% to −0.7% and the EF bias of the proposed 30% LRMC+HD‐PROST method is reduced from −8.9% to −6.9%. Bland–Altman plots comparing EF measurements using this “matched slice” approach are included in supporting information Figure S4D‐F.

Future work will consider whether the current motion field quality limitations can be overcome by the implementation of more‐advanced motion estimation methods. For example, several deep learning‐based techniques have recently been proposed^52^, ^53^, ^54^, ^55^ including LAPNet,^54^ which estimates non‐rigid motion directly from k‐space, and GRAFT,^55^ which enforces temporal smoothness and leverages additional information provided by the neighboring frames of the moving frame.

Another limitation of the cine comparison is that the conventional cine images were acquired with only 16 frames (single slice) or 20 frames (multi‐slice), in order to match the 16 frames used in the 3D approach. To compare cardiac function evaluation more accurately, a fuller comparison between the 3D approach and a standard 2D cine with more frames is needed in future studies.

The 3.3‐min scan time of the proposed method compares favorably not only to the 11‐min time of the previous motion‐resolved reconstruction^37^ but also to existing simultaneous whole‐heart $T_{1}$ and $T_{2}$ mapping techniques at a single cardiac phase,^32^, ^33^, ^34^, ^35^ which have reported scan times in the range of 7–9 min,^32^, ^33^, ^34^ or, in the case of the study by Guo et al.,^35^ 1.4 min, although this was achieved with 8‐mm‐thick slices and 2‐mm spacing between slices.

Additionally, while the results are pre‐contrast, the framework also has the potential to be applied post‐contrast and used in the generation of synthetic LGE images.^56^, ^57^, ^58^

A limitation of the proposed framework is the current long computational time. Using 30% of the acquired data, the LRI+HD‐PROST reconstruction at all 16 cardiac phases took ∼8 h, with an additional ∼2 h required for the LRMC+HD‐PROST reconstruction of each cardiac phase. Future improvements including code optimization, GPU‐based implementations, and the use of deep‐learning networks to replace certain steps could provide the necessary acceleration for clinical feasibility.

Currently, the proposed technique has only been validated in a small and healthy sample size (11 subjects). A larger study including patients with myocardial disease is thus warranted.

## 5 CONCLUSIONS

In this study, non‐rigid cardiac motion correction was incorporated into a free‐running framework for the simultaneous acquisition of 3D whole‐heart $T_{1}$ and $T_{2}$ myocardial maps and cine images with isotropic spatial resolution, enabling the acceleration of the scan time from 11 to 3.3 min. Three‐dimensional $T_{1}$ and $T_{2}$ maps and cine images with good image quality were generated for 11 healthy subjects. The proposed 3D radial sequence and LRMC+HD‐PROST reconstruction is thus a promising step toward a quick, simple, and comprehensive whole‐heart CMR exam.

## FUNDING INFORMATION

The authors acknowledge financial support from the BHF PG/18/59/33955, EPSRC EP/V044087/1, EP/P001009/, EP/P032311/1, and EPSRC EP/P007619, Wellcome EPSRC Centre for Medical Engineering (NS/A000049/1), Fondecyt 1210637 and 1210638, Millennium Institute for Intelligent Healthcare Engineering iHEALTH ICN2021_004.

## Supporting information




**Figure S1**. Mid‐ventricular short‐axis slices of the 3D second‐singular‐contrast images produced by different reconstruction methods and utilizing different amounts of k‐space data for a second representative subject.
**Figure S2**. Mid‐ventricular short‐axis slices of the 3D $T_{1}$ maps produced by different reconstruction methods and utilizing different amounts of k‐space data for a second representative subject. The mean and standard deviation of the $T_{1}$ value in the septum region are given in the corner of each panel.
**Figure S3**. Mid‐ventricular short‐axis slices of the 3D $T_{2}$ maps produced by different reconstruction methods and utilizing different amounts of k‐space data for a second representative subject. The mean and standard deviation of the $T_{2}$ value in the septum region are given in the corner of each panel.
**Figure S4**. Bland–Altman plots comparing the ejection fraction (EF) measured from the 3D LRMC+HD‐PROST and LRI+HD‐PROST reconstructions with that measured from the conventional multi‐slice 2D cine. (A–C) The 3D‐reconstruction EF is measured via the manual segmentation of every 2‐mm‐thick slice covering the left ventricle. (D–F) Every fourth 2‐mm‐thick slice (specifically, those matching an 8‐mm‐thick slice from the 2D multi‐slice cine) is used.
**Figure S5**. Bland–Altman plots comparing end‐systolic volume (ESV), end‐diastolic volume (EDV), and ejection fraction (EF) measured from LRMC+HD‐PROST reconstructions using 30% of the acquired data, 100% of the acquired data, or a mixed reconstruction utilizing 30% of the acquired data but the motion fields estimated from the LRI+HD‐PROST reconstruction with 100% of the acquired data (11 subjects).

**Video S1**. Video of the mid‐short‐axis 2D cine and the 3D cine at three slices for each of three slice orientations for one representative subject.
