# Supplementary material for: Free‐running 3D whole‐heart T1 and T2 mapping and cine MRI using low‐rank reconstruction with non‐rigid cardiac motion correction
Source: Magn Reson Med. 2022 Oct 5;89(1):217–32. doi: 10.1002/mrm.29449 (PMC9828568; doi:10.1002/mrm.29449)
Supplement: Supplementary file 1 — Figure S1. Mid‐ventricular short‐axis slices of the 3D second‐singular‐contrast images produced by different reconstruction methods and utilizing different amounts of k‐space data for a second representative subject. Figure S2. Mid‐ventricular short‐axis slices of the 3D T1 maps produced by different reconstruction methods and utilizing different amounts of k‐space data for a second representative subject. The mean and standard deviation of the T1 value in the septum region are given in the corner of each panel. Figure S3. Mid‐ventricular short‐axis slices of the 3D T2 maps produced by different reconstruction methods and utilizing different amounts of k‐space data for a second representative subject. The mean and standard deviation of the T2 value in the septum region are given in the corner of each panel. Figure S4. Bland–Altman plots comparing the ejection fraction (EF) measured from the 3D LRMC+HD‐PROST and LRI+HD‐PROST reconstructions with that measured from the conventional multi‐slice 2D cine. (A–C) The 3D‐reconstruction EF is measured via the manual segmentation of every 2‐mm‐thick slice covering the left ventricle. (D–F) Every fourth 2‐mm‐thick slice (specifically, those matching an 8‐mm‐thick slice from the 2D multi‐slice cine) is used. Figure S5. Bland–Altman plots comparing end‐systolic volume (ESV), end‐diastolic volume (EDV), and ejection fraction (EF) measured from LRMC+HD‐PROST reconstructions using 30% of the acquired data, 100% of the acquired data, or a mixed reconstruction utilizing 30% of the acquired data but the motion fields estimated from the LRI+HD‐PROST reconstruction with 100% of the acquired data (11 subjects). [file MRM-89-217-s001.pdf]

1 | DERIVATION OF EQUATIONS 4 AND 5

In the motion-resolved encoding operator defined in Equation 3, the dictionary-compression matrix for the qth cardiac phase, U_{rq}, is applied in k-space despite the dictionary relating to the signal evolution of pixel-values in image space. This is possible since, as has been previously demonstrated (see, for example, references 21 and 23), the Fourier transform and dictionary compression operations commute.

To similarly perform the dictionary compression operation in k-space in the motion-corrected case (Equation 5), we must also show that the dictionary-compression operation commutes with the non-rigid motion distortion operator M_q. Due to the size and structure of our matrices, U_{rq} and $(A_q F_q S)$ do not strictly commute. However, by restructuring the matrices using the same elements an equivalent definition of the encoding operator can be given as

$$E = \sum_q W(A_q F_q S) U_r M_q. \tag{7}$$

In this formulation, $W \in \mathbb{R}^{KN_c \times KN_c}$ and $M_q \in \mathbb{R}^{Nr \times Nr}$ are unchanged from Equation 5, but now $(A_q F_q S) \in \mathbb{C}^{KN_c \times KN}$ and $U_r \in \mathbb{R}^{KN \times Nr}$. We now seek to show that U_r and M_q effectively commute (and give identical results regardless of the order in which the operations are applied). As before, however, we note our matrices will not strictly commute due to their size and structure.

We begin by considering that U_r is arranged as $K \times r$ array of $N \times N$ blocks, and each of these blocks is a scalar matrix with the value of each of the diagonal elements being one of the $K \times r$ weights from the original (before restructuring) dictionary-compression matrix. That is,

$$U_r = \begin{bmatrix} \begin{pmatrix} u_{1,1} & 0 & \cdots & 0 \\ 0 & u_{1,1} & \cdots & 0 \\ \vdots & \vdots & \ddots & \vdots \\ 0 & 0 & \cdots & u_{1,1} \end{pmatrix} & \begin{pmatrix} u_{1,2} & 0 & \cdots & 0 \\ 0 & u_{1,2} & \cdots & 0 \\ \vdots & \vdots & \ddots & \vdots \\ 0 & 0 & \cdots & u_{1,2} \end{pmatrix} & \cdots & \begin{pmatrix} u_{1,r} & 0 & \cdots & 0 \\ 0 & u_{1,r} & \cdots & 0 \\ \vdots & \vdots & \ddots & \vdots \\ 0 & 0 & \cdots & u_{1,r} \end{pmatrix} \\ \begin{pmatrix} u_{2,1} & 0 & \cdots & 0 \\ 0 & u_{2,1} & \cdots & 0 \\ \vdots & \vdots & \ddots & \vdots \\ 0 & 0 & \cdots & u_{2,1} \end{pmatrix} & \begin{pmatrix} u_{2,2} & 0 & \cdots & 0 \\ 0 & u_{2,2} & \cdots & 0 \\ \vdots & \vdots & \ddots & \vdots \\ 0 & 0 & \cdots & u_{2,2} \end{pmatrix} & \cdots & \begin{pmatrix} u_{2,r} & 0 & \cdots & 0 \\ 0 & u_{2,r} & \cdots & 0 \\ \vdots & \vdots & \ddots & \vdots \\ 0 & 0 & \cdots & u_{2,r} \end{pmatrix} \\ \vdots & \vdots & \ddots & \vdots \\ \begin{pmatrix} u_{K,1} & 0 & \cdots & 0 \\ 0 & u_{K,1} & \cdots & 0 \\ \vdots & \vdots & \ddots & \vdots \\ 0 & 0 & \cdots & u_{K,1} \end{pmatrix} & \begin{pmatrix} u_{K,2} & 0 & \cdots & 0 \\ 0 & u_{K,2} & \cdots & 0 \\ \vdots & \vdots & \ddots & \vdots \\ 0 & 0 & \cdots & u_{K,2} \end{pmatrix} & \cdots & \begin{pmatrix} u_{K,r} & 0 & \cdots & 0 \\ 0 & u_{K,r} & \cdots & 0 \\ \vdots & \vdots & \ddots & \vdots \\ 0 & 0 & \cdots & u_{K,r} \end{pmatrix} \end{bmatrix} = \begin{bmatrix} u_{1,1}\mathbf{I} & u_{1,2}\mathbf{I} & \cdots & u_{1,r}\mathbf{I} \\ u_{2,1}\mathbf{I} & u_{2,2}\mathbf{I} & \cdots & u_{2,r}\mathbf{I} \\ \vdots & \vdots & \ddots & \vdots \\ u_{K,1}\mathbf{I} & u_{K,2}\mathbf{I} & \cdots & u_{K,r}\mathbf{I} \end{bmatrix}, \tag{8}$$

where $\mathbf{I}$ is the $N \times N$ identity.

Now, the operator E is applied to the multi-singular-contrast image vector $\boldsymbol{\rho}$ which is $rN \times 1$ and contains each of the N-long singular-contrast image vectors concatenated together. As the motion distortion for phase q is the same for each contrast, and is

applied to each contrast independently, M_q is block diagonal, with the $N \times N$ distortion matrix (labelled $\tilde{M}_q$ here for convenience) repeated diagonally r times as follows:

$$M_q = \begin{bmatrix} \tilde{M}_q & \mathbf{0} & \cdots & \mathbf{0} \\ \mathbf{0} & \tilde{M}_q & \cdots & \mathbf{0} \\ \vdots & \vdots & \ddots & \vdots \\ \mathbf{0} & \mathbf{0} & \cdots & \tilde{M}_q \end{bmatrix}. \tag{9}$$

The product of U_r and $\tilde{M}_q$ is thus

$$\begin{aligned} U_r M_q &= \begin{bmatrix} u_{1,1}\mathbf{I} & u_{1,2}\mathbf{I} & \cdots & u_{1,r}\mathbf{I} \\ u_{2,1}\mathbf{I} & u_{2,2}\mathbf{I} & \cdots & u_{2,r}\mathbf{I} \\ \vdots & \vdots & \ddots & \vdots \\ u_{K,1}\mathbf{I} & u_{K,2}\mathbf{I} & \cdots & u_{K,r}\mathbf{I} \end{bmatrix} \begin{bmatrix} \tilde{M}_q & \mathbf{0} & \cdots & \mathbf{0} \\ \mathbf{0} & \tilde{M}_q & \cdots & \mathbf{0} \\ \vdots & \vdots & \ddots & \vdots \\ \mathbf{0} & \mathbf{0} & \cdots & \tilde{M}_q \end{bmatrix} \\ &= \begin{bmatrix} u_{1,1}\mathbf{I}\tilde{M}_q & u_{1,2}\mathbf{I}\tilde{M}_q & \cdots & u_{1,r}\mathbf{I}\tilde{M}_q \\ u_{2,1}\mathbf{I}\tilde{M}_q & u_{2,2}\mathbf{I}\tilde{M}_q & \cdots & u_{2,r}\mathbf{I}\tilde{M}_q \\ \vdots & \vdots & \ddots & \vdots \\ u_{K,1}\mathbf{I}\tilde{M}_q & u_{K,2}\mathbf{I}\tilde{M}_q & \cdots & u_{K,r}\mathbf{I}\tilde{M}_q \end{bmatrix} \\ &= \begin{bmatrix} \tilde{M}_q\left(u_{1,1}\mathbf{I}\right) & \tilde{M}_q\left(u_{1,2}\mathbf{I}\right) & \cdots & \tilde{M}_q\left(u_{1,r}\mathbf{I}\right) \\ \tilde{M}_q\left(u_{2,1}\mathbf{I}\right) & \tilde{M}_q\left(u_{2,2}\mathbf{I}\right) & \cdots & \tilde{M}_q\left(u_{2,r}\mathbf{I}\right) \\ \vdots & \vdots & \ddots & \vdots \\ \tilde{M}_q\left(u_{K,1}\mathbf{I}\right) & \tilde{M}_q\left(u_{K,2}\mathbf{I}\right) & \cdots & \tilde{M}_q\left(u_{K,r}\mathbf{I}\right) \end{bmatrix} \\ &= \begin{bmatrix} \tilde{M}_q & \mathbf{0} & \cdots & \mathbf{0} \\ \mathbf{0} & \tilde{M}_q & \cdots & \mathbf{0} \\ \vdots & \vdots & \ddots & \vdots \\ \mathbf{0} & \mathbf{0} & \cdots & \tilde{M}_q \end{bmatrix} \begin{bmatrix} u_{1,1}\mathbf{I} & u_{1,2}\mathbf{I} & \cdots & u_{1,r}\mathbf{I} \\ u_{2,1}\mathbf{I} & u_{2,2}\mathbf{I} & \cdots & u_{2,r}\mathbf{I} \\ \vdots & \vdots & \ddots & \vdots \\ u_{K,1}\mathbf{I} & u_{K,2}\mathbf{I} & \cdots & u_{K,r}\mathbf{I} \end{bmatrix} \\ &= \hat{M}_q U_r, \end{aligned} \tag{10}$$

where $\hat{M}_q$ is, like M_q, a block diagonal matrix containing repetitions of $\tilde{M}_q$, but now contains K repetitions and is thus a $KN \times KN$ matrix.

Therefore, while U_r and M_q do not strictly commute, we see that it is equivalent to apply the motion distortion ($\tilde{M}_q$) either before U_r is applied (to each of the r singular contrasts) or after U_r is applied (to each of the time-point images). Hence, the order of operations presented in Equation 7 is valid, and, utilising the previously-established commutativity of U_r with $(A_q F_q S)$, Equation 5 is also valid.

2 | ADDITIONAL FIGURES

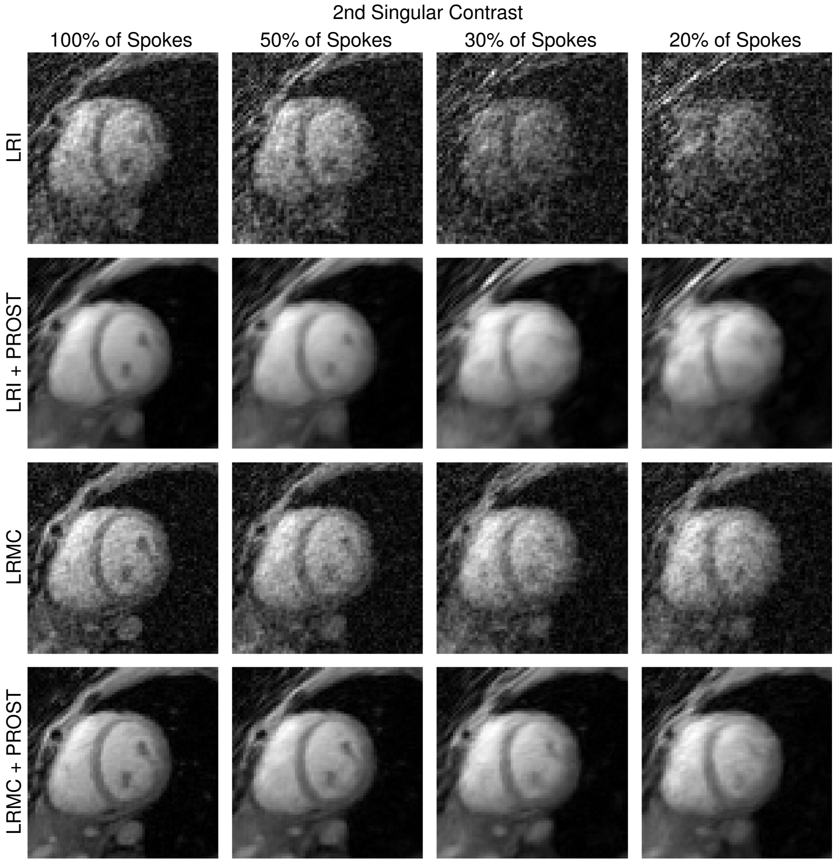

FIGURE S1 Mid-ventricular short-axis slices of the 3D 2nd-singular-contrast images produced by different reconstruction methods and utilising different amounts of k-space data for a second representative subject.

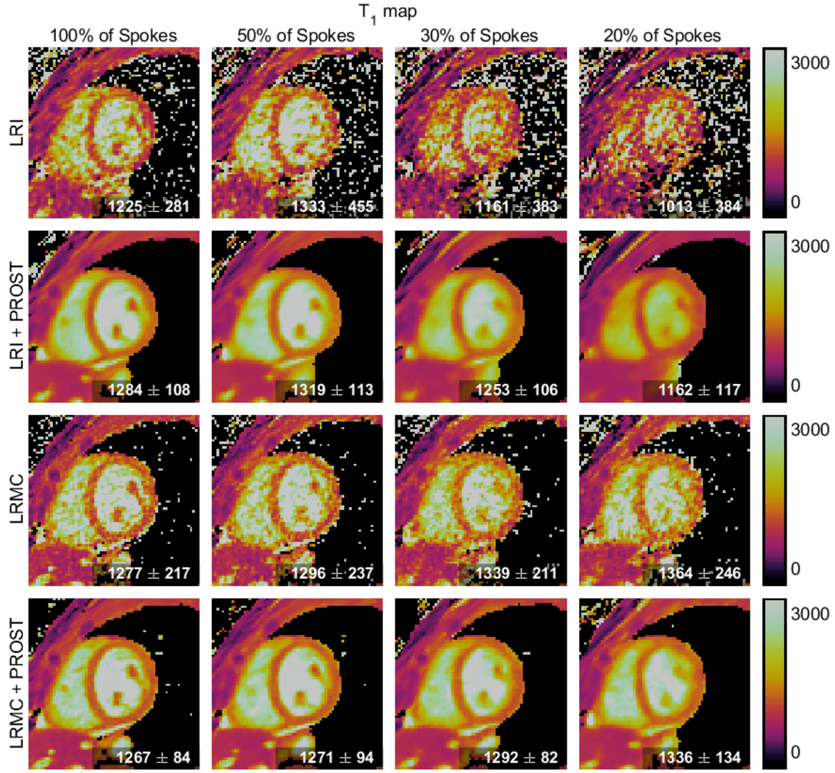

FIGURE S2 Mid-ventricular short-axis slices of the 3D T_1 maps produced by different reconstruction methods and utilising different amounts of k-space data for a second representative subject. The mean and standard deviation of the T_1 value in the septum region is given in the corner of each panel.

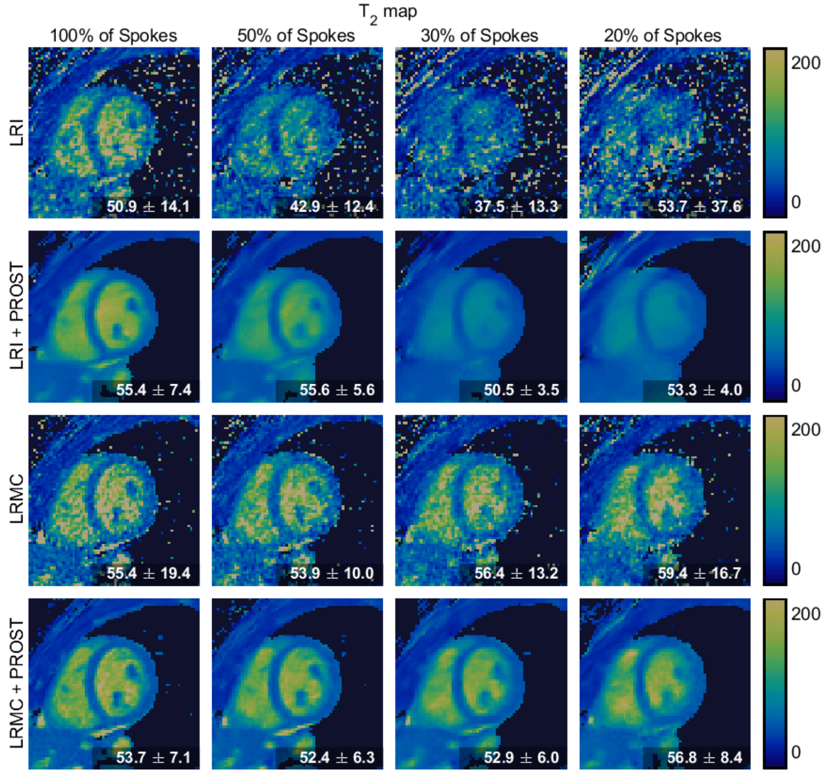

FIGURE S3 Mid-ventricular short-axis slices of the 3D T_2 maps produced by different reconstruction methods and utilising different amounts of k-space data for a second representative subject. The mean and standard deviation of the T_2 value in the septum region is given in the corner of each panel.

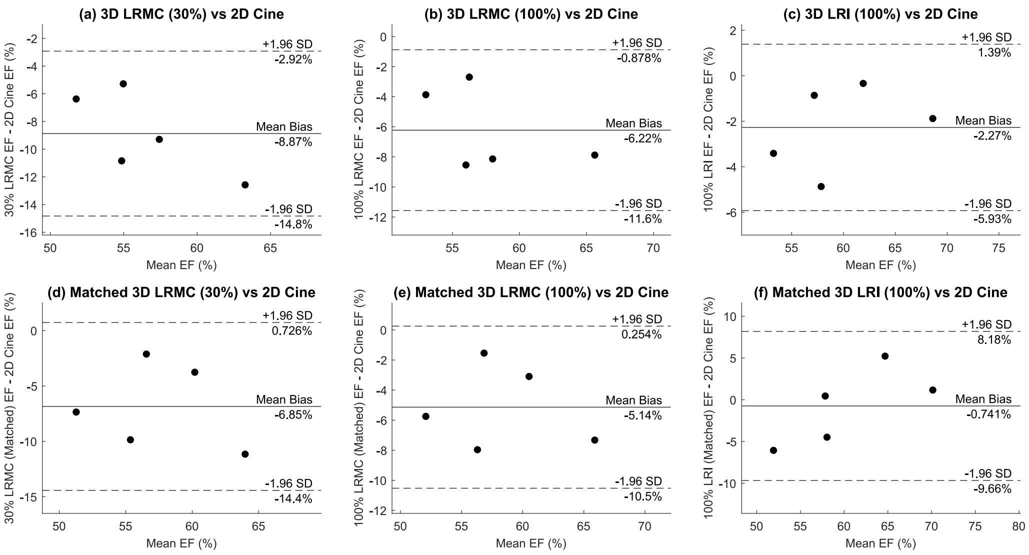

FIGURE S4 Bland-Altman plots comparing the ejection fraction (EF) measured from the 3D LRMC+HD-PROST and LRI+HD-PROST reconstructions with that measured from the conventional multi-slice 2D cine. In (a)-(c), the 3D-reconstruction EF is measured via the manual segmentation of every 2-mm-thick slice covering the left ventricle. In (d)-(f), only every fourth 2-mm-thick slice (specifically, those matching an 8-mm-thick slice from the 2D multi-slice cine) is used.

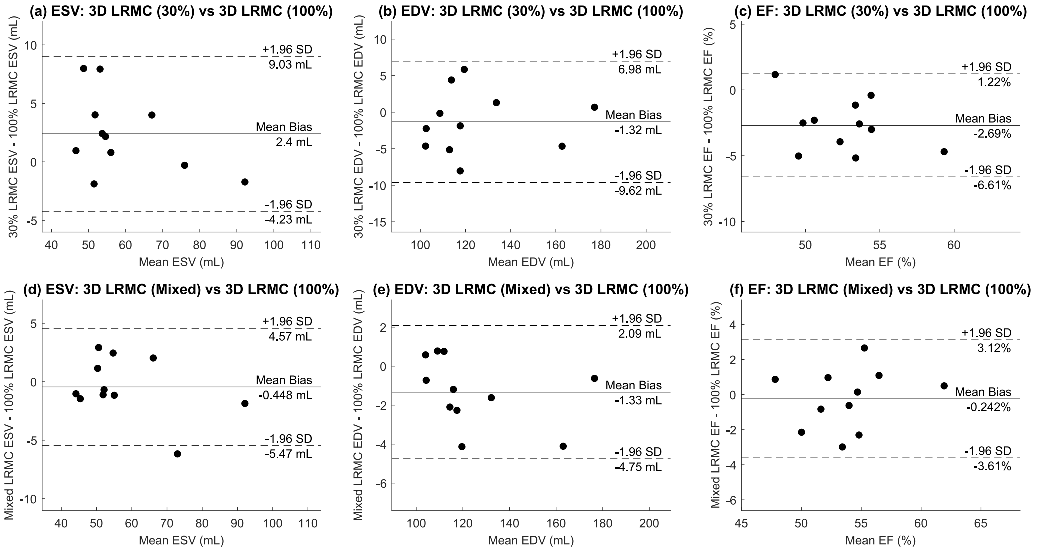

FIGURE S5 Bland-Altman plots comparing end-systolic volume (ESV), end-diastolic volume (EDV) and ejection fraction (EF) measured from LRMC+HD-PROST reconstructions using 30% of the acquired data, 100% of the acquired data or a mixed reconstruction utilising 30% of the acquired data but the motion fields estimated from the LRI+HD-PROST reconstruction with 100% of the acquired data (11 subjects).
